# Analysis of Bone Mineral Density (BMD) and Associated Risk Factors: A Single Center Study

**DOI:** 10.22038/aojnmb.2025.83172.1588

**Published:** 2026

**Authors:** Mahjabin Nobi Khan, Pabitra Kumar Bhattacharjee, Muhammad Farhan Muhtasim, Mohammad Ajijul Hoq, Noor-E-Amrin Alim, Mohammad Sazzad Hossain, Abdullah Al Persi, Anjuman Ara Akhter

**Affiliations:** Institute of Nuclear Medicine and Allied Sciences (INMAS), Bangladesh Atomic Energy Commission (BAEC), Chattogram, Bangladesh

**Keywords:** DXA, Parity, Rural, Urban, Diabetes A B S T R A C T

## Abstract

**Objective(s):**

Due to its prevalence worldwide, osteoporosis is regarded as a significant public health issue. Numerous risk factors can contribute to the development of osteoporosis, leading to bone fractures. This study aimed to determine the association of parity, age, gender, origin, and pre-existing clinical conditions with low bone density of people residing in the Southeastern region of Bangladesh.

**Methods:**

This retrospective study included a cohort of 628 individuals seeking bone mineral density (BMD) assessments using Dual Energy X-ray Absorptiometry (DXA). Association of BMD with five contributing factors (age, gender, origin, parity of the female participants and pre-existing clinical conditions i.e., hypertension, diabetes) was studied.

**Results:**

This study included 84% female and 16% male participants. The mean BMD was found significantly lesser in older participants (aged >50 years) (*P*=.0.001)for both sites of lumbar spine (LS) and femoral neck (FN)). It also varied significantly in both skeletal sites, depending on the gender (*P*<0.001and *P*=0.027respectively). Rural participants were found with lower BMD than urban one (*P*<0.001and *P*=0.037respectively). BMDs of LS and FN were found to have significant negative correlations with age (-0.201 and -0.280) and parity (-0.317 and -0.236). Diabetic participants were found to have higher bone density in this study (*P*=0.002 and* P*<0.001) compared with the non-diabetic. Multivariate regression analysis also revealed statistically significant associations of BMD with gender (*P*<0.001), age (*P*<0.001), and origin (*P*=0.001) for the lumbar spine and with gender (*P=0.002*) and age (*P*<0.001) for the femoral neck. A significant decrease in mean BMD was also found in multiparous (parity ≥3) females compared to low parity (parity 1-2) and nulliparous (parity 0) females (*P*<0.001 and *P*=0.048 respectively for LS and FN).

**Conclusion:**

A proactive approach to prevent osteoporosis in the study population involves a meticulous investigation of its etiological factors and addressing them precedently. Therefore, it is recommended to consider BMD as a routine test for prevention, early detection, and to minimize the sequelae of osteoporosis.

## Introduction

 Osteoporosis globally affects approximately 8.9 million people every year (1). This metabolic bone disease usually affects individuals with advancing age. Osteoporosis, characterized by porous bone, leads to the deterioration of bone mass, resulting from the loss of essential minerals like calcium, magnesium, and phosphate (2). Several findings showed that after the age of fifty, one in every three women and one in every five men are at the risk of being diagnosed with osteoporotic fractures (3, 4). As this affliction occurs, the intricate structure of bones declines, resulting in a higher prevalence of fractures, particularly in the hip, spine, forearm, and proximal humerus (4). Research indicates that osteoporosis is the leading cause of hip fractures globally, with one osteoporotic fracture occurring every three seconds (2). In 2010, the highest number of osteoporotic patients was found in Asia (55%) and the researchers predicted that the number would be doubled by 2040 (5).

 Like any other Asian country, in Bangladesh osteoporosis is extensively common in elderly population and especially postmenopausal women. The risk of osteoporosis has been found to be significantly associated with older age and the same study has indicated an increased occurrence of osteoporosis in patients with chronic illness and multi-morbidity (6). In Bangladesh, varying levels of vitamin D deficiency—contributing to reduced bone density and osteoporosis—have been found to be significantly associated with both age and gender (7). Other studies have also revealed that pregnancy, birth, lactation, diabetes (DM), and hypertension (HTN) are also associated with osteoporosis (8-11). Pregnancy and lactation cause an elevation in calcium mobilization and resorption, causing calcium deficiency of the mothers, which in the later stage of life can harm the bone health. Multiple pregnancies and birth (multi-parity) has been found to lower the BMD of women in various studies (8). 

 Moreover, at the later period of lactation, bone loss occurs along with decreasing BMD (12). A higher risk of bone fractures has been found in diabetic patients compared to nondiabetic (13). Diabetic patients with bone fractures showed slower wound healing and even a risk of increased mortality (14).

 In Bangladesh, a substantial number of patients visit nuclear medicine centers located throughout the country to measure bone mineral density (BMD) and evaluate their bone health, aiming to rule out osteoporosis. Using Dual-energy X-ray Absorptiometry (DXA), bone mineral density of the lumbar spine, bilateral femoral neck, and total hip are measured in these centers (6). According to the definition of the World Health Organization (WHO), the value of T-score is an indicator of the status of skeletal mineralization, while the values indicate -2.5≤ T-score ≤-1 and T-score <-2.5 meaning osteopenia and osteoporosis respectively (15). The number of patients, being recommended by various physicians to measure their bone mineral density, is increasing day by day in our country. This indicates rising awareness among general people and hints at the worsening of their bone health too. At this point, the risk factors and reasons behind the deteriorating condition of bone, demand crucial exploration.

## Methods

### Study design and population

 This retrospective study included bone mineral density data of 628 participants, who were referred to the Institute of Nuclear Medicine and Allied Sciences (INMAS), Chattogram, Bangladesh, for DXA scan. These patients, mostly referred by specialized physicians, expressed concerns regarding the symptoms of osteoporosis. 

 Patients who attended this institute for their scans within the time span of 01 January 2022 to 30 June 2023 (18 months) were considered for the study. The inclusion criteria included adult participants who came for bone mineral densitometry, and the exclusion criteria included pregnant and child participants. The clinical histories of each of the participants were obtained concomitantly after obtaining their verbal consent. All of the acquired information was tabulated for further analysis. 

 Additionally, the obstetric history of the female participants was taken regarding their number of pregnancies & offspring. The participants were assured that the privacy of their personal information would be completely maintained. Furthermore, the data would not be identifiable during and after the analysis for the study.

 For research purposes, the mentioned data were accessed between January and February 2024. In the current study, the bone mineral density of the lower lumbar spine (LS) and femur neck (FN) was measured. An association with low bone mineral density was observed with five different variables, which are age, gender, pre-existing disease conditions (i.e. diabetes, hypertension), place of residence (urban and rural), and parity (the number of births reported by the female participants). For this study, we considered, women having up to two children as ‘low parity’, more than two children as ‘multiparous’ and having no child as ‘nulliparous’. Participants’ information was obtained through a concise interview process. In case of the pre-existing clinical conditions, previous medical history of diabetes and hypertension was considered for this study, with a majority of the participants taking medication. Though the patients had self-reported diabetes, the type of the diabetes was not considered for analysis.

 The bone density was measured using a Dual-energy X-ray absorptiometry (DXA) densitometer, MEDIX-DR, Medi Link. In this process, the X-ray beam is imposed on the body of the patient and depending on the amount of absorbed X-ray, bone density is measured. As per the WHO’s (World Health Organization) definition, the machine-generated T-scores and values of bone density (gm/cm²) of the participants were analyzed to delineate their bone health (S1 Table). One skilled technician performed the DXA scan. Thereafter, an experienced nuclear medicine physician interpreted the results following the WHO criteria (S1 Table) and finally, a scientist made the study-design, gathered the whole data and analyzed it throughout this study.

**S1 Table:** According to WHO (World Health Organization), the definitions of osteoporosis and osteopenia from DXA scan results [14]

T-Score(Bone density scale)	
Normal bone mass	>-1
Osteopenia	-1 to -2.5
Osteoporosis	<-2.5

### Ethics Statement

 All participants provided their verbal consent regarding to this study. The study got the ethical approval from the Ethical Review Committee of Chittagong Medical College, Chattogram-4000, Bangladesh (memo no.59.27.0000.013.19.PG. 2024.009/305).

### Statistical analysis

 Data collection, statistical analysis, and data presentation were carried out using Microsoft Excel and IBM SPSS v20. Continuous variables were calculated by ‘mean±standard deviation’. 

 Association between continuous variables was assessed by Pearson correlation coefficient: two-tailed; *P*≤0.01 was considered statistically significant. Dependency tests between categorical variables were done by the Chi-squared test of independence. In that instance, the osteoporosis and osteopenia together were considered as low bone density (LBD) group. 

 Hypothesis test was done by independent sample t-test to compare the mean difference of quantitative data between two groups of sample population and multivariate regression analysis was also done to find the association of the independent variables with bone mineral density.

## Results

 The fundamental characteristics of 628 participants considered in this study are displayed in the S2 Table. The mean age of the participants was 57.75±11.93 years with a minimum of 20 years and a maximum of 90 years. The measurements of bone mineral densities (BMD) in lower lumbar spine (LS) and femoral neck (FN) resulted in the mean values of 0.78±0.18 gm/cm² and 0.84±0.18 gm/cm² respectively. The mean T score of lumbar spine was found to be -1.84±1.75 while that of the femoral neck was -0.62±1.48.

**S2 Table:** Fundamental characteristics of sample population

	Mean (SD)	Minimum	Maximum
Age	57.75±11.93 years	20 years	90 years
BMD (LS)	0.78±0.18 gm/cm²	0.33 gm/cm²	1.53 gm/cm²
BMD (FN)	0.84±0.18 gm/cm²	0.13 gm/cm²	1.39 gm/cm²
T- score (LS)	-1.84±1.75	-6.4	5.5
T- score (FN)	-0.62±1.48	-16	3.3

 Out of the 628 participants, the study included 526 (84%) of female and 102 (16%) of male participants. The bone mineral density status of male and female participants were separately studied (S3 Table). In case of lumbar spine, 240 (38%) participants were found with osteoporosis, 222 (35%) with osteopenia and 166 (27%) with normal bone mineral density. 

 Among the cases of osteoporosis in lumbar spine, 207 (86%) of females and 33 (14%) of males were affected. Similarly, 187 (84%) of females and 35 (16%) of males were diagnosed with osteopenia. Lastly, 132 (80%) of females and 34 (20%) of males were found to have normal bone mineral density in the same skeletal site of lumbar spine.

 In femoral neck, 51 (8%) participants were diagnosed with osteoporosis including 39 (76%) of female and 12 (24%) of male participants. Additionally, among 197 (31%) participants, 161 (82%) were females and 36 (18%) were males, all of whom were found to have osteopenia. Finally, 380 (61%) participants were found with normal bone density in femoral neck, comprising 326 (86%) females and 54 (14%) males (S3 Table).

**S3 Table:** Gender specific BMD status of study population

Gender	Lumbar spine			Femoral neck		
	Osteoporosis	Osteopenia	Normal	Osteoporosis	Osteopenia	Normal
Female (526, 84%)	207 (86%)	187 (84%)	132 (80%)	39 (76%)	161 (82%)	326 (86%)
Male (102, 16%)	33 (14%)	35 (16%)	34(20%)	12 (24%)	36 (18%)	54 (14%)
Total	240 (38%)	222 (35%)	166 (27%)	51 (8%)	197 (31%)	380 (61%)

 Therefore, to assess whether there is any significant difference in bone mineral density between female and male groups of participants, an independent sample t-test was done. The test showed that the mean BMD of the lumbar spine is lower in the female participants (0.76±.0.17 gm/cm²) than the male (0.85±0.21gm/cm²), a mean difference of 0.09 (95% CI, 0.05 to 0.13) gm/cm², *t*(626)=4.619, *P*<0.001. Similarly, in the case of the femoral neck, the mean BMD of females (0.84±0.17 gm/cm²) is less than males (0.88±0.20 gm/cm²), a mean difference of 0.04 (95% CI, 0.004 to 0.08) gm/cm², *t*(626)=2.216, *P*=0.027 (S4 Table). 

**S4 Table:** Analysis of the mean difference of bone mineral density of lumbar spine and femoral neck depending on the gender of the study population (Independent sample t-test)

Skeletal site	Gender	Mean BMD± SD (gm/cm²)	Mean Difference (gm/cm²)	t-score & *P*-value
Lumbar spine	Female	0.76±0.17	0.09	t(626)=4.619
	Male	0.85±0.21		*P*<0.001
Femoral neck	Female	0.84±0.17	0.04	t(626)=2.216
	Male	0.88±0.20		*P*=0.027

 The sample population was divided into two separate groups depending on age, with one encompassing individuals over the age of 50 and the other consisting of those up to 50. The bone mineral density was observed for those two groups. For both the skeletal sites of lumbar spine and femoral neck, a notable elevation of osteoporosis and osteopenia is observed among individuals aged above 50 years (S5 Table). The mean BMD of the participants is significantly lower in the older age group for both the skeletal sites. The mean BMD of lumbar spine is lesser in the group of participants aging >50 years (0.76±0.18 gm/cm²) than that of the participants aging ≤ 50 years (0.83±0.17 gm/cm²), a mean difference of .07 (95% CI, 0.04 to 0.10) gm/cm², *t*(626)=4.447, *P*<0.001. In the same way, mean BMD of femoral neck is lesser in the group of participants aging >50 years (0.82±0.16 gm/cm²) than that of the participants aging ≤ 50 years (0.91±0.20 gm/cm²), a mean difference of .09 (95% CI, 0.06 to 0.12) gm/cm², *t*(626)= 5.585, *P*<0.001 (S5 Table).

**S5 Table:** Comparative analysis of bone mineral density status among distinct age groups

Skeletal sites	Age groups (**years**)	BMD status			Mean BMD ± SD (gm/cm²)	Mean Difference (gm/cm²)	t-score & *P*-value
		Osteoporosis	Osteopenia	Normal			
Lumbar spine	≤ 50	44 (26%)	57 (34%)	68 (40%)	0.83±0.17	0.07	*t*(626)=4.447
	> 50	196 (43%)	165 (36%)	98 (21%)	0.76±0.18		*P*<0.001
Femoral neck	≤ 50	9 (5%)	37 (22%)	123 (73%)	0.91±0.20	0.09	*t*(626)=5.585
	> 50	42 (9%)	160 (35%)	257 (56%)	0.82±0.16		*P*<0.001

**Figure 1:**
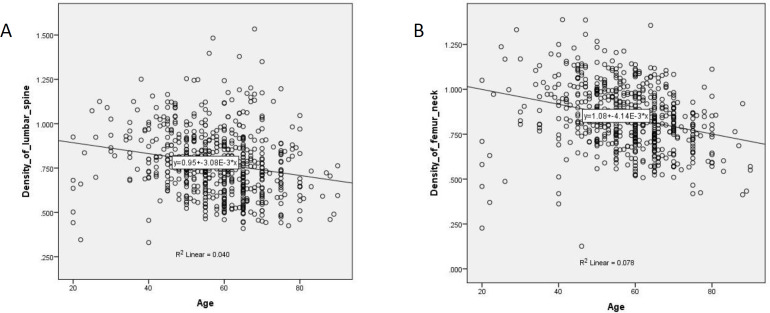
Scatter diagram showing correlation between Age and BMD of two different skeletal sites (Scatter-Plot). (**A**) Scatter diagram showing significant negative correlation between age and the bone mineral density of lumbar spine (left), (**B)** Scatter diagram showing significant negative correlation between age and the bone mineral density of femoral neck (right)

 Correlation between the age and bone density of two different skeletal sites: lumbar spine and femoral neck were also analyzed (Figure 1). Significant negative correlations between age and bone mineral densities were found in both cases (Pearson’s correlation coefficients of -0.201 and -0.280 for lumbar spine and femoral neck respectively; correlations are significant at the 0.01 level; two-tailed test).

 Place of residence of the participants was also taken into account for the study. Around 52% (324) and 48% (304) of the study population respectively from urban and rural areas were studied. An independent sample t-test was conducted to assess whether there were any mean differences in bone mineral density (BMD) of lumbar spine and femoral neck among the participants living in rural and urban area (S6 Table). The test results showed that the mean bone mineral density of lumbar spine is 

greater for the participants of urban area (0.80±0.19 gm/cm²) than that of the rural area (0.75±0.17 gm/cm²), a mean difference of 0.05 (95% CI, 0.02 to 0.05) gm/cm², *t*(626)=3.56, *P<0.001*. The findings similarly indicated that the average femoral neck bone mineral density is higher in the participants of urban area (0.86±0.17 gm/cm²) compared to those from rural area (0.83±0.18 gm/cm²), a mean difference of 0.03 (95% CI, 0.002 to 0.06) gm/cm², *t*(626)=2.09, *P*=0.037.

**S6 Table:** Origin specific BMD status of study population (Independent sample t-test)

Skeletal site	Origin	Mean BMD± SD (gm/cm²)	Mean Difference (gm/cm²)	t-score & *P*-value
Lumbar spine	Urban	0.80±.19	0.05	*t*(626)=3.56
	Rural	0.75±.17		*P*<0.001
Femoral neck	Urban	0.86±.17	0.03	*t*(626)=2.09
	Rural	0.83±.18		*P*=0.037

 While considering pre-existing disease conditions, 32% (204) and 49% (308) participants were found to have diabetes (DM) and hypertension (HTN) respectively. Moreover, it was observed that 23% (147) of sample population had both diabetes and hypertension. To determine the significance of the differences, a two-sample t-test was conducted and results are shown in S7 Table.

 The analysis showed that the average bone mineral density of lumbar spine is higher in diabetic patients (0.81±0.17 gm/cm²) compared to non-diabetic group of participants (0.76±0.19 gm/cm²), with a mean difference of 0.05 (95% CI, 0.02 to 0.08) gm/cm², *t*(626)=3.04, *P*=0.002. Likewise, in femoral neck, BMD is higher in diabetic group (0.88±0.17 gm/cm²) than that of non-diabetic (0.82±0.18 gm/cm²), showing mean difference of 0.06 (95% CI, 0.03 to 0.09) gm/cm², *t*(626)=3.845, *P<0.001*. Although the mean difference in BMD was significantly higher in diabetic patients compared to nondiabetic individuals, multiple regression analysis revealed that diabetes mellitus (DM) was significantly associated with other variables: gender, age and, hypertension for both LS and FN (gender: *P*-value 0.050; age: *P*-value 0.050; HTN: *P*<0.001 for both LS and FN).

 In case of hypertension, the statistical analysis showed that the mean BMD of lumbar spine is 0.78±0.17 gm/cm² for hypertensive and 0.77±0.19 gm/cm² for non-hypertensive patients, with mean difference of .01 (95% CI, -0.02 to 0.04) gm/cm², *t*(626)=0.694, *P*=0.488. When it comes to femoral neck, the BMD is 0.85±0.17 gm/cm² for hypertensive and 0.84±0.18 gm/cm² for non-hypertensive patients, having mean difference of 0.01 (95% CI, -0.01 to 0.04) gm/cm², *t*(626)=1.049, *P*=0.295.

**S7 Table:** Comparative study of BMD depending on the pre-existing disease condition

Pre-existing disease conditions		Skeletal sites	Mean BMD±SD (gm/cm²)	Mean Difference (gm/cm²)	t-score & *P*-value
Diabetes	Yes	Lumbar spine	0.81±0.17	0.05	*t*(626)=3.04
	No		0.76±0.19		*P*=0.002
	Yes	Femoral neck	0.88±0.17	0.06	*t*(626)=3.845
	No		0.82±0.18		*P<0.001*
Hypertension	Yes	Lumbar spine	0.78±0.17	0.01	*t*(626)=.694
	No		0.77±0.19		*P*=0.488
	Yes	Femoral neck	0.85±0.17	0.01	*t*(626)=1.049
	No		0.84±0.18		*P*=0.295

 A multivariate regression analysis was done to determine the independent effect of variables on BMD and the association between the variables. Through this analysis the associations of gender, age, origin, disease conditions of diabetes, hypertension and both diabetes and hypertension with the BMD of lumbar spine and femur neck were done. The analysis showed statistically significant associations with gender (*P*<0.001), age (*P*<0.001), and origin (*P*=0.001) for the lumbar spine and similarly with gender (*P*=0.002) and age (*P<0.001*) for the femoral neck. However, nearly 11% (*P<0.001*, ANOVA) and 12% (*P<0.001*, ANOVA) of the variance in bone mineral density of LS and FN were explained respectively by these variables altogether. Variables related to BMD for both LS and FN included gender and age. Neither analysis found any association with diabetes, hypertension, and both diabetes and hypertension (S8 Table, S9 Table).

**S8 Table:** Variables associated with BMD in the study population for the lumbar spine by multivariate regression analysis

**Variables**	**Unstandardized Coefficients**		**Standardized Coefficients**	**t**	**Sig. (** ***P*** **-value)**
	**B**	**Std. Error**	**Beta**		
(Constant)	0.918	0.057		16.093	*<0.001*
Gender	0.101	0.019	0.204	5.373	*<0.001*
Age	-0.004	0.001	-0.233	-6.016	*<0.001*
Origin	0.047	0.014	0.129	3.422	0.001
Diabetes	-0.021	0.025	-0.053	-0.824	0.410
Hypertension	-0.006	0.017	-0.015	-0.327	0.744
Both diabetes & hypertension	-0.046	0.031	-0.106	-1.467	0.143

**S9 Table:** Variables associated with BMD in the study population for the femoral neck by multivariate regression analysis

Variables	Unstandardized Coefficients		Standardized Coefficients	t	Sig. (*P*-value)
	B	Std. Error	Beta		
**(Constant)**	1.145	0.055		20.915	*<0.001*
**Gender**	0.057	0.018	0.119	3.151	0.002
**Age**	-0.005	0.001	-0.311	-8.086	*<0.001*
**Origin**	0.025	0.013	0.071	1.887	0.060
**Diabetes**	-0.044	0.024	-0.116	-1.823	0.069
**Hypertension**	-0.017	0.017	-0.048	-1.009	0.313
**Both diabetes & hypertension**	-0.023	0.030	-0.056	-0.774	0.439

 Out of 526 female participants, the data on parity were accessible for 310 individuals. Female participants with low parity, multiparity, and nulliparity were found to be 23% (71), 75% (233), and 2% (6), respectively.

**Figure 2:**
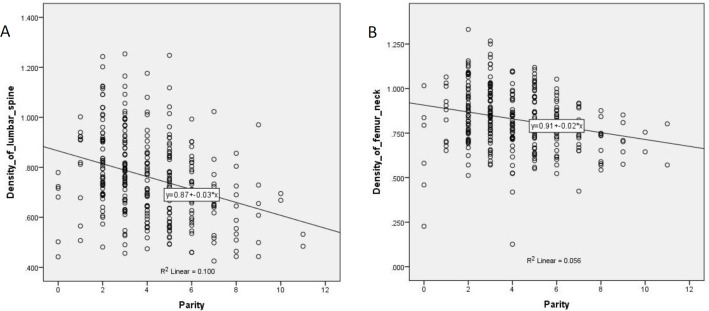
Correlation between parity and BMD of two different skeletal sites (Scatter-Plot). **A** Scatter diagram showing significant negative correlation between parity and the bone mineral density of lumbar spine of the female participants (**left**), **B**) Scatter diagram showing significant negative correlation between parity and the bone mineral density of femoral neck of the female participants (right)

Pearson’s correlation was studied between parity and bone mineral density (Figure. 2). 

 Between parity and bone mineral densities, very high significant negative correlations were found for both the skeletal sites, lumbar spine and femoral neck. The results indicated that the BMD is more negative with the increasing number of parity. For lumbar spine and femoral neck, the Pearson’s correlation coefficients are -0.317and -0.236 respectively; correlations are significant at the 0.01 level; two tailed test. 

 The bone mineral densities of both the skeletal sites were also compared among the multiparous and non-multiparous (parity ≤2) female participants (S10 Table). The average BMD of the lumbar spine in multiparous female is significantly lesser (0.74±0.16 gm/cm²) than that of non-multiparous group (0.82±0.17 gm/cm²), with a mean difference of 0.08 (95% CI, 0.03 to 0.12) gm/cm², *t*(308)=3.559, *P*<0.001. Likewise, the average BMD of femoral neck in multiparous female participants is also lesser (0.82±0.16 gm/cm²) than that of the other (0.86±0.18 gm/cm²), with a mean difference of 0.04 (95% CI, 0.0004 to 0.08) gm/cm², *t*(308)= 1.986, *P*=0.048 (S10 Table).

**S10 Table:** Comparative analysis of bone mineral density of multiparous female with non-multiparous female participants

Skeletal sites	Parity	Mean BMD±SD (gm/cm²)	Mean Difference (gm/cm²)	t-score & *P*-value
Lumbar spine	Multiparous	0.74±0.16	0.08	*t*(308)=3.559
	Non-Multiparous	0.82±0.17		*P*<0.001
Femoral neck	Multiparous	0.82±0.16	0.04	*t*(308)= 1.986
	Non-Multiparous	0.86±0.18		*P*=0.048

## Discussion

 Low bone mineral density, consequently resulting in osteoporosis, is a common multifactorial disease that impose potential effect on morbidity and mortality of major population worldwide (16). Age, gender, pre-existing clinical conditions like hypertension, diabetes, sedentary lifestyles etc. are found to be the contributing factors for low bone density in different study population (11, 16). In this study, the association of a few of these specific factors with the bone mineral density was studied, which showed differential results. As one of the most important factors, age has been found to influence bone density. With advancing age, the bone density decreased significantly in the study population. Individuals aged over 50 years demonstrated a significantly reduced BMD compared to the participants at or below 50 years. Body composition of males and females also variedly contributes to the bone density. While the average BMD in males is higher than in females, it was observed that male bone density declined with age (17, 18). 

 The current study indicates that the mean difference of BMD is significant between males and females. In line with the other analyses, for both the sites of LS and FN, the average BMD is lower in female participants than that of males. Moreover, the area of residence can also have a potential effect on health status. A meta-analysis involving fifteen articles from eleven distinct populations made conflicting decisions about the effect of urbanization on the variation of bone density (19). In high-income countries, the BMD was found higher for urban people, however, in case of the middle and low-income countries, the results are mixed (19). Research revealed a greater occurrence of reduced bone density among elderly women residing in rural areas compared to their younger counterparts in urban locales (20). Similarly, this analysis also suggests that there is a significant difference of mean bone mineral density between theparticipants living in rural and urban areas. For both the skeletal sites, the mean BMDs of both the LS and FN, are lower in rural people than that of urban.

 The relationship of pre-existing clinical conditions such as diabetes and hypertension with BMD varied across different studies indicating significant association with bone mineral density (9, 10). The findings from the meta-analysis indicated a decline in BMD among individuals with type 1 diabetes, while BMD was notably elevated in those with type 2 diabetes, when compared to the BMD of healthy individuals (21, 22). In our analysis, for both the skeletal sites: lumbar spine and femoral neck, the mean BMD is significantly higher in diabetic patients than that of non-diabetics. Multiple regression analysis further revealed significant association of diabetes mellitus with other independent variables gender, age and hypertension. These covariates may partly explain the elevated BMD observed in the diabetic group. These findings recommend a further comprehensive investigation into understanding the impact of diabetes mellitus on bone mineral density. However, the older and the hypertensive patients often exhibit lower bone density and they have a higher risk of developing of osteoporosis (16). Few studies showed that essential hypertension could reduce the BMD of the lumbar spine and femoral neck, which is also significant in Asian population (11). Hypertension and stroke were identified as two of the major risk factors for bone fractures, whereas acute myocardial infarction, atrial fibrillation, and deep venous thromboembolism were observed as less prominent contributors to this risk (23). 

 However, the hypertensive patients of our study showed no significant difference of BMD in comparison with non-hypertensive ones. Besides these common confounders, the bone mineral density of female participants was specially analyzed, focusing on their parity. 

 However, there is a limitation of this study, which is the incomplete information about the parity of female participants. Only 310 out of 526 female participants gave the information about their childbirth, which were considered for analysis in this study. Female participants were found to have significantly decreased bone density with the increasing parity. The mean bone mineral density of multiparous women was also found significantly lower in both LS and FN than that of others. Study regarding the influence of parity on the bone density was previously done. In case of post-menopausal women, association between bone mineral density and increased number of parity was found negative by other researchers (24). 

 Another study involving the young and middle-aged Arabic women revealed that nulliparous women has higher BMD than that of multiparous (9, 10). Our study came out with similar findings. In short, the multiparous women are prone to develop poor bone growth.

 The bone mineral density was assessed using Dual Energy X-ray Absorptiometry (DXA) in this study. This method enabled quick, noninvasive bone mineral measurements ensuring minimum radiation exposure to the participants. Various evidence based scientific improvements and technological advancements have enhanced the efficiency and affordability of this procedure for patients (25). Widespread availability and ease of access have made DXA, one of the most widely used technique for measurements of bone density in clinical trials and epidemiological studies.

## Conclusion

 Scientific analysis of the bone mineral density of our study population exhibits a significant correlation with the age of the participants. This study also demonstrates marked disparities of bone density between participants living in urban and rural areas, indicating a vital impact of their standards of living on bone health. The awareness and education of self-care of urban people can be the contributing factor for their better bone condition compared to that of rural. During the study of pre-existing clinical conditions, such as diabetes, it raised an inquiry regarding the better bone health observed in diabetic patients compared to non-diabetic individuals. For better understanding of these results, further comprehensive studies are required in this context. Low bone mineral density is also a concerning issue for women having multiple children as they grow poor bone with advancing age. Considering the findings from our retrospective data analysis, we suggest measuring BMD as a routine test to prevent, detect early and minimize the sequela of osteoporosis. 

 Further large-scale study is recommended to quantify the extent of bone density related complications and its association with multiple risk factors.
